# Assessment of Eye Fatigue Caused by 3D Displays Based on Multimodal Measurements

**DOI:** 10.3390/s140916467

**Published:** 2014-09-04

**Authors:** Jae Won Bang, Hwan Heo, Jong-Suk Choi, Kang Ryoung Park

**Affiliations:** Division of Electronics and Electrical Engineering, Dongguk University, 26 Pil-dong 3-ga, Jung-gu, Seoul 100-715, Korea; E-Mails: bangjw@dgu.edu (J.W.B.); gjghks@dgu.edu (H.H.); jjongssuk@dgu.edu (J.-S.C.)

**Keywords:** eye fatigue, 3D display, multimodal measurement, remote gaze tracking system

## Abstract

With the development of 3D displays, user's eye fatigue has been an important issue when viewing these displays. There have been previous studies conducted on eye fatigue related to 3D display use, however, most of these have employed a limited number of modalities for measurements, such as electroencephalograms (EEGs), biomedical signals, and eye responses. In this paper, we propose a new assessment of eye fatigue related to 3D display use based on multimodal measurements. compared to previous works Our research is novel in the following four ways: first, to enhance the accuracy of assessment of eye fatigue, we measure EEG signals, eye blinking rate (BR), facial temperature (FT), and a subjective evaluation (SE) score before and after a user watches a 3D display; second, in order to accurately measure BR in a manner that is convenient for the user, we implement a remote gaze-tracking system using a high speed (mega-pixel) camera that measures eye blinks of both eyes; thirdly, changes in the FT are measured using a remote thermal camera, which can enhance the measurement of eye fatigue, and fourth, we perform various statistical analyses to evaluate the correlation between the EEG signal, eye BR, FT, and the SE score based on the T-test, correlation matrix, and effect size. Results show that the correlation of the SE with other data (FT, BR, and EEG) is the highest, while those of the FT, BR, and EEG with other data are second, third, and fourth highest, respectively.

## 1. Introduction

The recent rapid development of 3D displays has resulted in the need for various types of 3D devices such as anaglyphs, passive and active glasses, and non-glasses device types. However, with the increasing prevalence of 3D displays, user eye fatigue has become an important health issue when one is viewing a 3D display. Eye fatigue caused by fatigue, eyestrain, and dizziness, *etc.* is usually induced by the discordance between accommodation and convergence when viewing 3D displays [1]. Many previous studies have therefore focused on measuring eye fatigue, which is classified into two categories: Single modality-based and multiple modality-based methods.

Single modality-based methods measure eye fatigue using a single modality such as the image features captured by a camera [2–5], and they use only bio-signals [6–12]. In previous studies, eye blinks were used to measure eye fatigue, and they were observed from images captured by a camera [2,4,5]. Other studies showed that visual fatigue could be measured by analyzing eye movements and eye blinks [3]. In one study [5], the authors quantitatively measured the eyestrain for a 3D display, and their measurements were based on the eye blinking rate (BR). In this case, they calculated the levels of various factors considering eye foveation modal and edge information.

In general, methods that involve the use of image features obtained by a camera have the advantage of causing less discomfort to the participants during experiments than methods involving the use of bio-signals, which require the attachment of cumbersome sensors to one's body in order to measure the bio-signals. In addition, bio-signals may contain noises caused by muscle movements. However, the speed with which conventional cameras (such as web-cams) can acquire images is usually lower than what is possible when acquiring bio-signals due to the larger volume of data to be transmitted from the camera to the computer.

In previous studies, eye fatigue has therefore been measured by analyzing eye movements using electrooculography (EOG) signals [6]. Other studies showed that electroencephalograms (EEGs) that are based on an event-related potential (ERP) may be used to measure visual fatigue [7,9]. Chen *et al.* showed that the alpha, beta, and delta bands of EEG signals can be used to measure visual fatigue for 3D TV [8]. In another previous study, visual fatigue was evaluated by analyzing the user's electrocardiography (ECG) signals [10]. Qian *et al.* showed that the blink signals extracted from EEG data may indicate eye fatigue [11]. Mun *et al.* proposed a method for identifying the steady-state visually evoked potential (SSVEP) and ERP, which are linked to 3D cognitive fatigue for stereoscopic 3D displays using EEG signals [12]. In a previous study, they also measured eye fatigue when viewing 3D displays, and used ECGs and subjective evaluation (SE) [13].

With respect to multiple modality-based methods, eye fatigue has been measured using multiple sensors simultaneously [14,15]. Kim *et al.* proposed a method for measuring eye fatigue on a 3D display using ECG sensors, the galvanic skin response (GSR), and the skin temperature (SKT) with SE [14]. Eye fatigue when viewing 3D displays has also been measured using the power of the beta bands of EEGs and BRs considering a Bayesian network [15]. These methods have the advantage of usually being more accurate than the methods involving a single modality. However, the attachment of multiple sensors may cause much discomfort to the user, which can lead to incorrect eye fatigue measurements [14]. In addition, the accuracy enhancement of measurement of eye fatigue just by two modalities of EEG and BR is limited [15].

To overcome these problems of previous works, we propose a method of assessing eye fatigue caused by 3D displays based on multimodal measurements including EEG signals, eye BR, and facial temperature (FT) with SE both before and after watching a 3D display. To accurately measure the BR in a manner that is more convenient to the user, we implement a remote gaze-tracking system using a high-speed mega-pixel camera that can measure the BR of both eyes. Changes in the FT are measured using a remote thermal camera that can enhance the measurement of eye fatigue. Table 1 shows summarized comparisons of previous and proposed methods for measuring eye fatigue.

The remainder of this paper is structured as follows: In Section 2, we describe the proposed system and the feature analysis. Then, the experimental setup and results are described in Section 3. Finally, we present the conclusions in Section 4.

## 2. Proposed Method for Measuring Eye Fatigue

### 2.1. Experimental Procedure for Proposed System and Method

**Figure 1.**
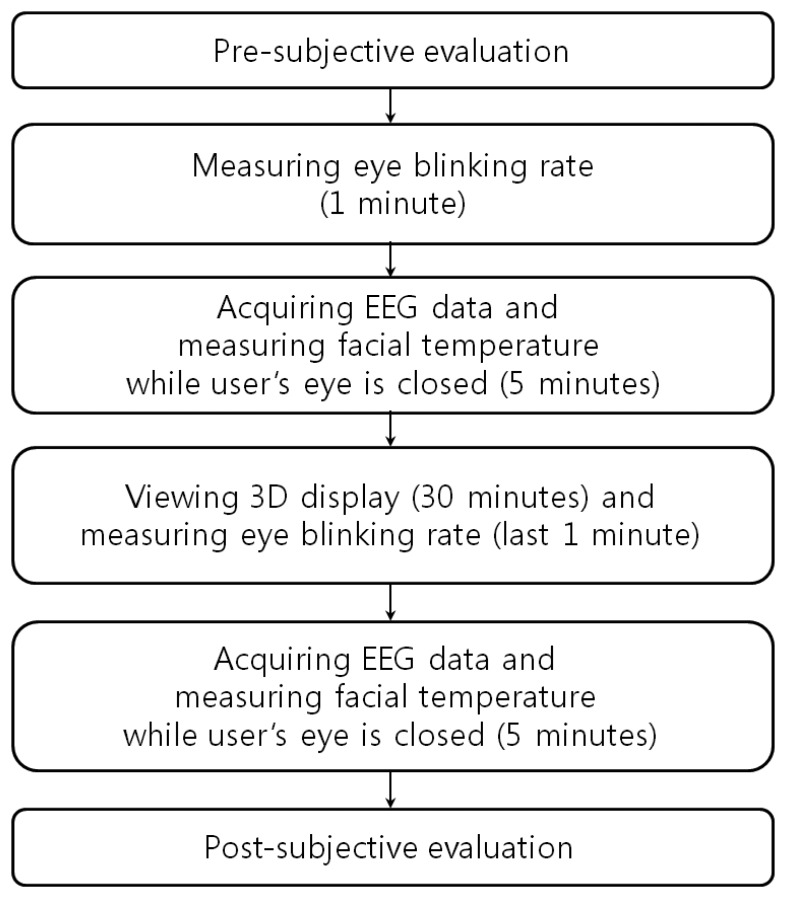
Experimental procedures used in our research.

Figure 1 shows the experimental procedures used in our research. First, a SE was carried out in order to check the subject's condition before watching the 3D display. In order to measure the natural blink of the subjects, the eye BR was measured for 1 min. before the user was made to watch the 3D display. Also, prior to watching the 3D display, the user's EEG data and FT were measured for 5 min. with eyes closed in order to minimize visual stimuli. The subjects then watched the 3D display for 30 min. The eye BR was measured for the last 1 min of watching in order to compare the variations in the eye BR immediately before and after watching the 3D display. After watching the display, the EEG data and FT were again measured for 5 min. while the user's eyes were closed in order to minimize visual stimuli, and these measurements were used to compare the variations in the EEG and FT data. A SE was carried out to check the subject's condition after watching the 3D display.

**Figure 2.**
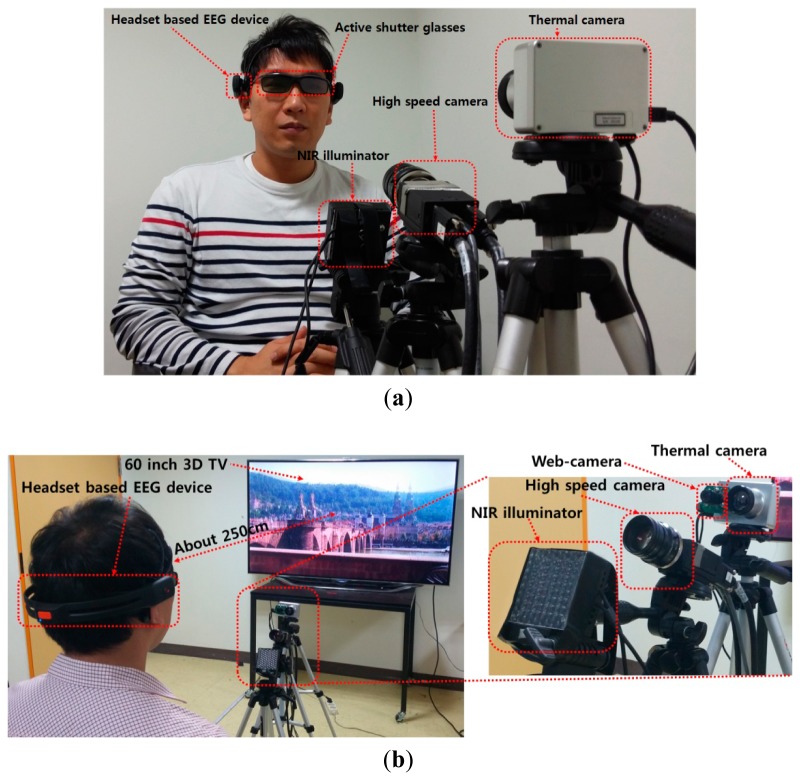
Proposed system for the assessment of eye fatigue. (**a**) Proposed experimental device; (**b**) Example of experimental environment.

As shown in Figure 2a, a user wears both a headset-based EEG device and active shutter glasses [16] for the experiment. As shown in Figure 2b, the user wearing shutter glasses and the EEG device watches 3D content shown on a 60-inch smart TV with a display resolution of 1920 × 1080 pixels. In order to assess eye fatigue, we measured the EEG signals, eye BR, and FT using the EEG device, high speed camera [17], and thermal camera [18], respectively.

**Figure 3.**
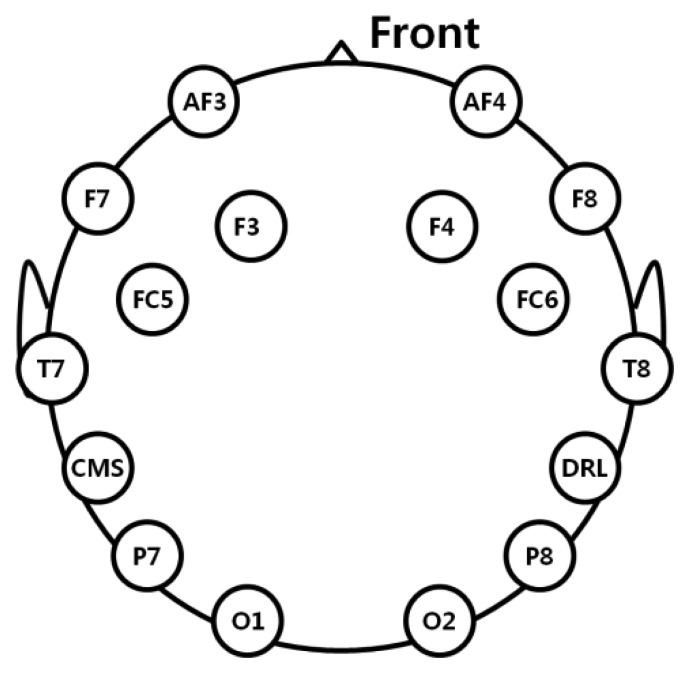
International 10–20 electrode placement system.

We used a commercial EEG device with a low-cost Emotiv EPOC headset that consists of 16 electrodes, including two reference nodes for measuring EEG signals [19], as shown in Figure 2. The placement of electrodes complies with the international 10-20 system like previous researches [20,21], as shown in Figure 3. EEG data were processed using a built-in digital 5th-order Sinc filter. The sampling rate of the Emotiv EPOC system was 128 Hz (128 samples/s) [20,22].

A high-speed camera of 4 mega pixels was used to measure the eye BR and to acquire both images of both eyes. The camera acquires images with a resolution of 2048 × 2048 pixels at a speed of 150 fps [17]. However, the actual acquisition speed of images is much lower than 150 fps because of the larger amount of data to be transmitted from the camera to the computer, and then to be written to the hard disk. In order to improve the image acquisition speed, a cropped image with 2048 × 512 pixels (including both eyes) is captured. To enhance the accuracy of the detection of the pupil region, a near infrared (NIR) illumination of 850 nm was used, as shown in Figure 2b, which does not dazzle the user's eye [23].

As shown in Figure 2, a thermal camera was used to measure the FT based on changes in the eye fatigue. The captured thermal image has a resolution of 320 × 240 pixels with 14 bits, and its capturing speed ranges from 50 to 60 fps. However, the actual capture speed of images is much lower than 50 fps because of the large amount of data to be transmitted from the camera to the computer, and then to be written by the hard disk. The temperature of the thermal camera ranges from −20 °C to 100 °C, and its accuracy is ±1 °C or ±1% [18]. As shown in Figure 2b, a commercial web-camera (Webcam C600) [24] was attached close to the thermal camera in order to locate the user's face and nose. Because it is difficult to find accurate positions of the face and nose in the thermal image, we used the positions of the face and nose in the web-camera image to define the areas of the face and nose in the thermal image. The web-camera captures an 800 × 600 pixel 24-bit image at a speed of 30 fps using the NIR illuminator. The NIR illuminator is used to detect the face and nose in a manner that is robust to illumination variations.

As shown in Figure 2b, the distance between the subject and the 3D TV is set at about 250 cm considering the guidelines for watching 3D TV [25]. The distance between the subject and the high-speed camera is about 80 cm, while the distance between the subject and the thermal camera is about 100 cm, and the distance between the subject and the NIR illuminator is about 60 cm.

### 2.2. Analysis of EEG Data for Measuring Eye Fatigue

To measure eye fatigue, we analyzed the beta band (13–30 Hz) of the EEG data because the power of EEG signals in the beta band is usually stronger when viewing 3D displays [7,10]. The EEG signals are determined based on the voltage levels measured from each electrode. The measured EEG signals are normalized by adjusting the DC level, and a further normalization of the min-max scale was performed to represent the EEG magnitude within the range −1 to 1. Then, the power of the beta band was calculated by performing a Fourier transform with a window length of 128 samples.

### 2.3. Analysis of BR for Measuring Eye Fatigue

Eye BR has previously been used in the measurement of eye fatigue. A higher eye fatigue is related to a higher eye BR [2,3,26]. Therefore, in our research, the eye BR was measured using a high-speed camera. In order to accurately measure the eye BR in a manner that is convenient to the user, we implemented a remote gaze-tracking system using a high-speed mega-pixel camera that can measure the eye blinks of both eyes.

**Figure 4.**
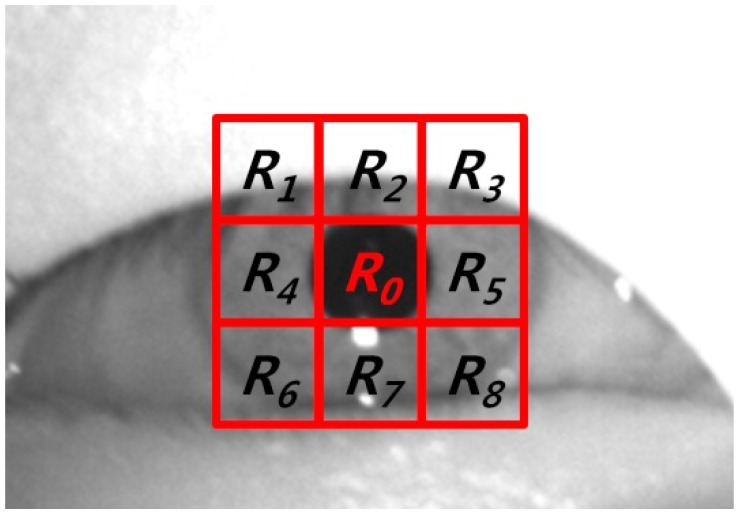
Example of sub-block-based template matching algorithm.

**Figure 5.**
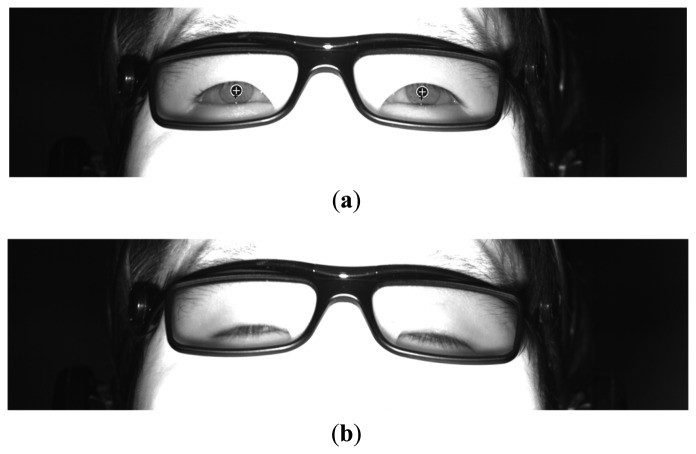
Example of measurement of eye blinking. (**a**) Open eyes; (**b**) Closed eyes.

With the captured image, the region of the corneal specular reflection is located using image binarization. Two areas of the eyes are detected based on the detected region of the corneal specular reflection using sub-block based template matching, which is based on the scheme of integral images in order to reduce the computational complexity [27]. The means of each sub-block (*R_0_* ∼ *R_8_*) are calculated as shown in Figure 4, where *R_0_* and *R_1_* ∼ *R_8_* represent the candidate pupil regions and its neighboring ones, respectively. The mean of the gray value of the pupil region is usually lower than those of the other regions. Therefore, the mean of the gray value of *R_o_* in the candidate pupil region is compared with the means of the gray values of *R_1_* ∼ *R_8_* in the neighboring regions, as shown in Figure 4. The sum of the difference of the mean values of *R_0_* and the neighboring regions (*R_1_* ∼ *R_8_*) is then calculated. In order to find the pupil position for which the sum of the difference value is a maximum, the 3 × 3 mask in Figure 4 moves by the overlapping of one pixel. Within the area detected by the sub-block-based template matching, the boundary of the pupil is located using the ellipse fitting method, as shown in Figure 5a. For closed eye, as shown in Figure 5b, the ellipse fitting method fails, whereas for opened eyes, the ellipse fitting method is successful. We are therefore able to differentiate between images with both open and closed eyes, and the eye BR is counted based on the number of images with closed eyes during a one-minute interval.

### 2.4. Analysis of Variation of FT for Measuring Eye Fatigue

Changes in the FT were measured using a remote thermal camera, which can enhance the measurement of eye fatigue. A web-camera [24] is attached beside the thermal camera because it is difficult to detect the facial features in the thermal image. Therefore, with the detected areas of the face and nose in the web-camera image, the areas of the face and nose are defined considering the image disparity between the web-camera and thermal camera. The degree of image disparity is obtained in advance by calibrating the camera.

**Figure 6.**
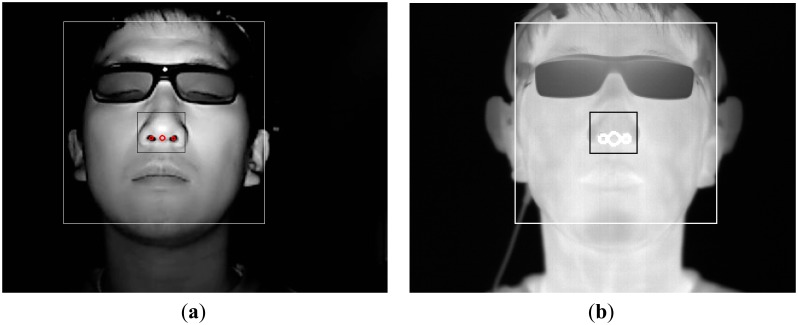
Example of detection of face and nose. (**a**) The detected regions of face and nose in the web-camera image; (**b**) The defined regions of the face and nose in the thermal camera image.

The web-camera captures the image with the NIR illuminator. The reason for using the NIR illuminator is to detect the face and nose in a manner that is robust to variations in illumination. As shown in Figure 6a, the detection of the face region is achieved by the adaptive boosting (Adaboost) method [28], and the center of both nostrils based on the detected face region is located using binarization. The regions of the face and center of the nose are defined in the thermal camera image based on the regions of the face and center of the nose in the web-camera image, as shown in Figure 6b.

**Figure 7.**
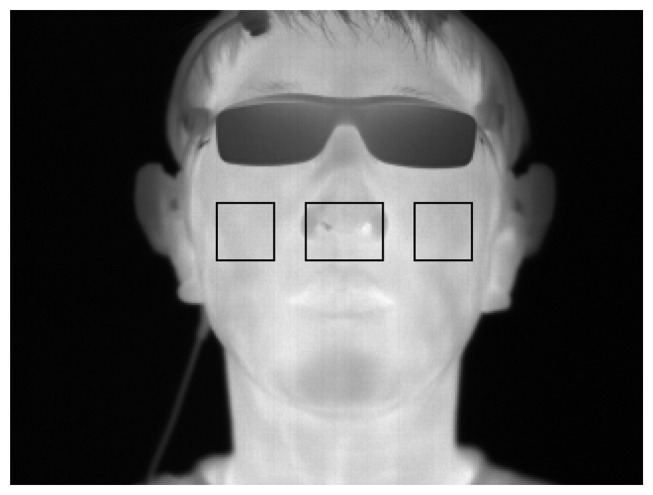
Example of the measurement region for variation of FT.

Based on the detected center of the nose, variations in the cheek regions (30 × 30) are analyzed for the measurement of eye fatigue, as shown in Figure 7.

## 3. Experimental Setup and Results

The data acquisition for the experiments was done using two desktop computers and a laptop computer. All of the EEG signal, eye image, and thermal image data were acquired simultaneously. The desktop computer that was used to acquire images of both eyes with a high-speed camera was equipped with a 3.07 GHz CPU (Intel (R) Core (TM) i7 CPU 950) and 6 GB RAM. The desktop computer used to acquire the EEG signals using the Emotiv EPOC headset was equipped with a 2.33 GHz CPU (Intel (R) Core (TM) 2 Quad CPU Q8200) and 4 GB RAM. The laptop computer used to acquire the web-camera and thermal camera images was equipped with a 2.50 GHz CPU (Intel (R) Core (TM) i5-2520M) and 4 GB RAM.

**Figure 8.**
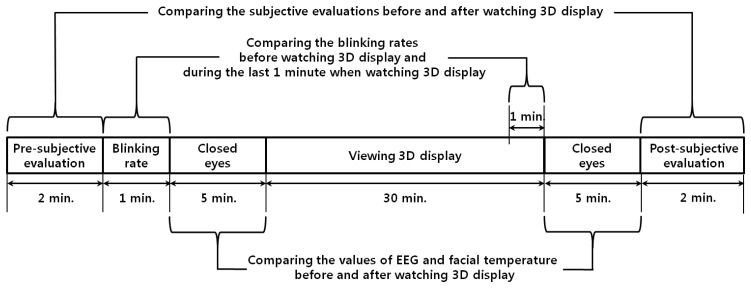
Experimental procedure.

The proposed algorithm for the measurement of eye fatigue was implemented as a C++ program using the Microsoft Foundation Class (MFC) and OpenCV library. A total of 15 subjects participated in the experiments (average age: 26.89, standard deviation: 1.96). The numbers of male and female are 12 and 3, respectively. We already obtained the written and informed agreements from each participant of our experiments. The luminance of the room was 321 lux., and the highest brightness of the display was 99.546 cd/m^2^. We used the sample video entitled “*Summer in Heidelberg*” for our experiments, which is mostly composed of landscape scenes as shown in Figure 2b, and we already obtained the permission from the video copyright owner [5,29]. In order to measure the rate of natural eye blink in the last 1 min of Figure 8, any artificial sign, indication or instruction for user's alertness was not given to each participant. There was no drowsy or dozing person in the experiments.

**Figure 9.**
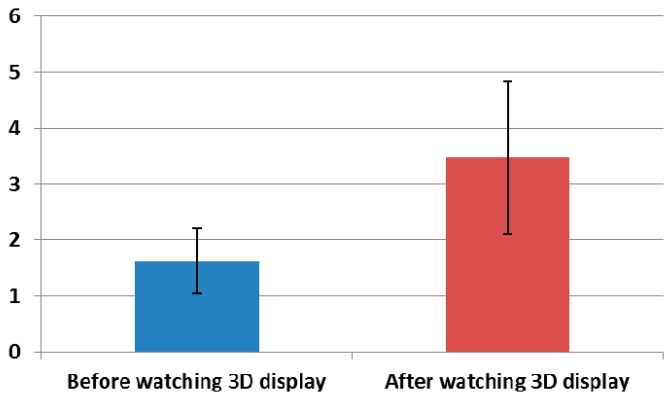
Comparison of SE scores before and after watching the 3D display.

The experimental procedure is presented in Figure 8. In order to enhance the accuracy of the eye fatigue assessment, changes in the EEG signal, eye BR, and FT were measured with SE both before and after watching the 3D display. In previous research [8], they measured the variations of EEG data caused by eye fatigue on 3D TV by using 16 electrodes on the whole area of head. They also measured the EEG data while a user is closing his eyes before and after watching 3D TV. We refer to this paper for our experimental design of measuring EEG signal using multiple electrodes while a user is closing his eyes. To compare the subject's state before and after watching the 3D display, SE was performed using a questionnaire form. The SE before and after watching the 3D display was performed based on the six questions in Table 2 using a 10-point scale (1: Not at all ∼ 10: Yes, very much). These questions were developed based on previous studies [5,30]. The average and standard deviation of the score obtained by SE of the 15 subjects before and after watching the 3D display are presented in Figure 9 and Table 3.

The average SE score after watching the 3D display is observe to be higher than that before watching the 3D display, as indicated by Figure 9 and Table 3. The statistical analysis was performed using an independent two-sample T-test [31], which is widely used as a statistical hypothesis test. The calculated p-value is 0.0001, which is less than the confidence level of 99% (0.01). The null-hypothesis (the two average scores of SEs before and after watching the 3D display are the same) is rejected, and the two scores of SE before and after watching the 3D display are significantly different [31].

**Figure 10.**
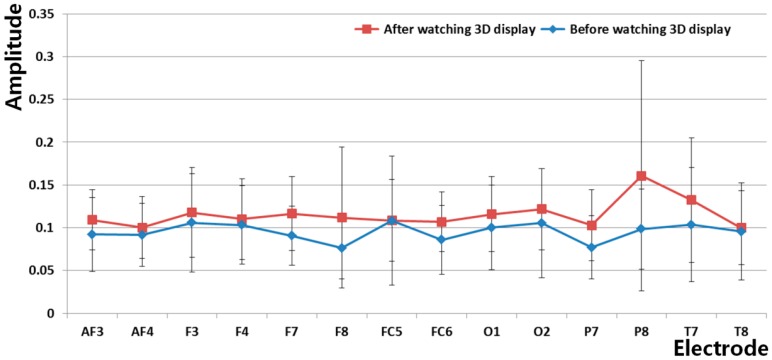
Comparisons of beta band of EEG data before and after watching 3D display.

Figure 10 and Table 4 show the measured amplitudes of the beta band of each electrode of the EEG signal before and after watching the 3D display. As shown in Figure 10, the amplitude of the beta band of each electrode is stronger after watching the 3D display than before watching the 3D display. The electrode that shows a significant difference before and after watching the 3D display was selected using the T-test, as shown in Table 4. The P7 of the lowest p-value (0.0795) of all the electrodes was selected, as shown in Table 4. Although the p-value of the P7 node is lowest as 0.0795, it does not show the significant difference with the confidence level of 95% or 99% because the p-value is larger than the thresholds for 95% (0.05) and 99% (0.01), respectively. This is because EEG signals contain noise that is caused by movements of the facial muscle. Nevertheless, we used the EEG signal of P7 node because its significant difference is relatively higher than those of other nodes, and the level of inter-correlations between each measurements of EEG, BR, FT, and SE score are measured as shown in Tables 8 and 9.

In total 16 electrodes are used in our EEG measurement device as shown in Figure 3, and the strength of attachment (on head) of some nodes of backside of head (P7, P8, O1, and O2 of Figure 3) is relatively lower than that of others due to the individual variations of head shape. Among them, the strength of attachment of P8 node is lowest, and the consequent variation of movement of P8 node on head is larger than others during experiment, which causes the large standard deviation value of Figure 10.

**Figure 11.**
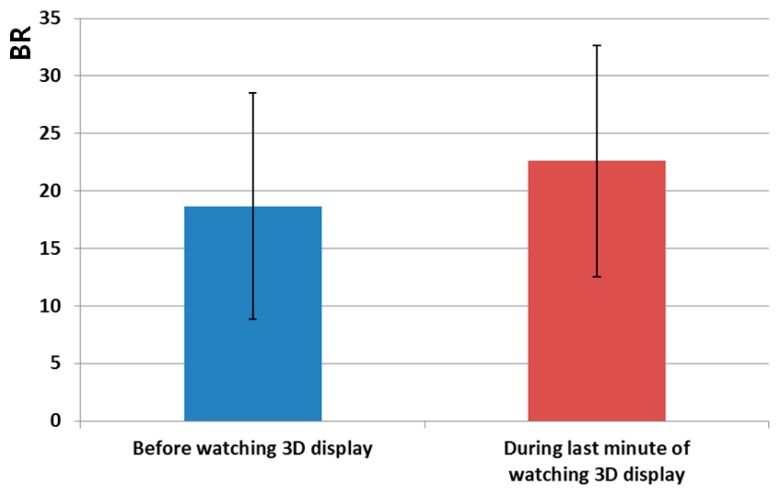
Comparison of BR before watching the 3D display and in the last 1 minute (of 30 min) of watching the 3D display.

Figure 11 and Table 5 show the measured eye BRs before watching the 3D display and during the last 1 min of watching the 3D display. As shown in Figure 11, the BR increased in the last 1 min of watching the 3D display compared to the value before watching the 3D display. The p-value is 0.2876, which is larger than confidence levels of 99% (0.01) or 95% (0.05). It is therefore difficult to say that there is a significant difference in the BR before watching the 3D display and in the last 1 min of watching the 3D display. The BR increased by 21.07% (100 × (22.6 – 18.667)/18.667) in the last 1 min of watching the 3D display relative to the value before watching the 3D display, as shown in Table 5.

**Figure 12.**
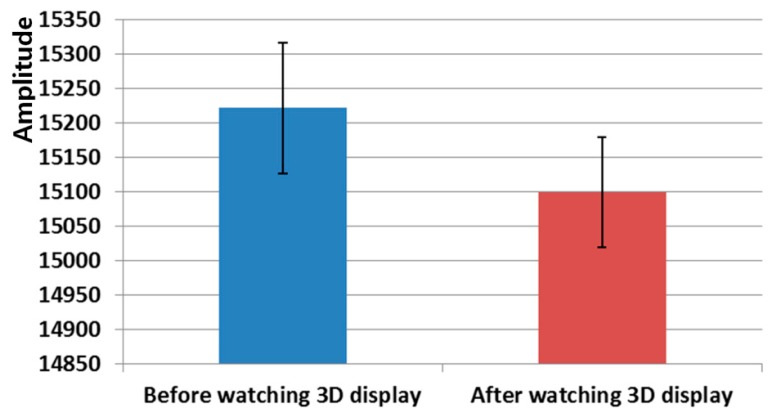
Comparison of FT amplitude before and after watching the 3D display.

As shown in Figure 12 and Table 6, the amplitude of the FT decreased after watching the 3D display compared to the value before watching the 3D display. The p-value is 0.00089, which is much less than the confidence level of 99% (0.01). We can therefore deduce that there is a significant difference in the FT amplitudes before and after watching the 3D display.

For the next analysis, we measured the difference between the amount of data before and after watching the 3D display using the effect size in descriptive statistics. In statistics, the effect size has been used to represent the power of an observed phenomenon, and it is generally accepted as a descriptive statistic [5,32].

**Figure 13.**
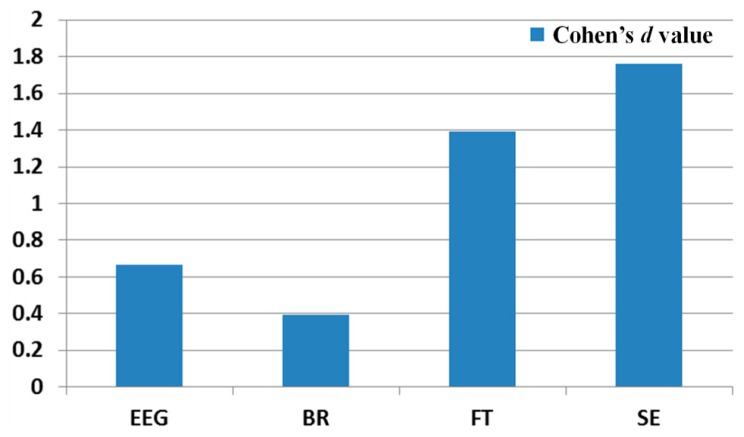
Calculated Cohen's *d* values of each measurement.

Based on previous research [33], we can define Cohen's *d* values of 0.2, 0.5, and 0.8 as small, medium, and large, respectively. In Table 7 and Figure 13, Cohen's *d* represents the difference between two means divided by the standard deviation of the data. By calculating Cohen's *d*, effect sizes of 0.2 to 0.3, around 0.5, and 0.8 to infinity may be considered as having “small,” “medium,” and “large” effects, respectively [5,32]. For example, in Table 7, Cohen's *d* of the EEG data before and after watching the 3D display is 0.6644, which is closer to 0.8 (large effect) than to either 0.2 (small effect) or to 0.5 (medium effect). Therefore, we deduce that the difference in the EEG data before and watching the 3D display has a large effect size. It is often the case that 0.5 is used among the values of around 0.5, and we also used 0.5 for the medium effect. Therefore, the disparity (0.1356) between 0.8 and 0.6644 is smaller than that (0.1644) between 0.6644 and 0.5, and we regard this case (0.6644) as the large effect size as shown in Table 7. As another example, Cohen's *d* for the BR before and in the last 1 min of watching the 3D display is 0.3958, which is closer to 0.5 than to either 0.2 or 0.8. Thus, we deduce that the difference in the BR before and in the last 1 min of watching the 3D display has a medium effect size. In the cases of the FT and SE, the effect sizes are large.

Based on the p-value and Cohen's *d* value, we find that the difference between the SEs before and after watching the 3D display is largest. In addition, the differences between FT, EEG, and BR are second, third, and fourth largest, respectively.

For the next analysis, we measured the correlation between two sets of data from among EEG, BR, FT, and SE data. To do this, we calculated the gradient, R^2^, and correlation value between two sets of data, as shown in Table 8. With the data distributed in 2-dimensional (2D) space, an optimal fitted line is obtained by linear regression, and the gradient and R^2^ value can be calculated. R^2^ represents the confidence level of the predicted regression line. If the regression line fits the data more reliably, the R^2^ value increases [5].

**Figure 14.**
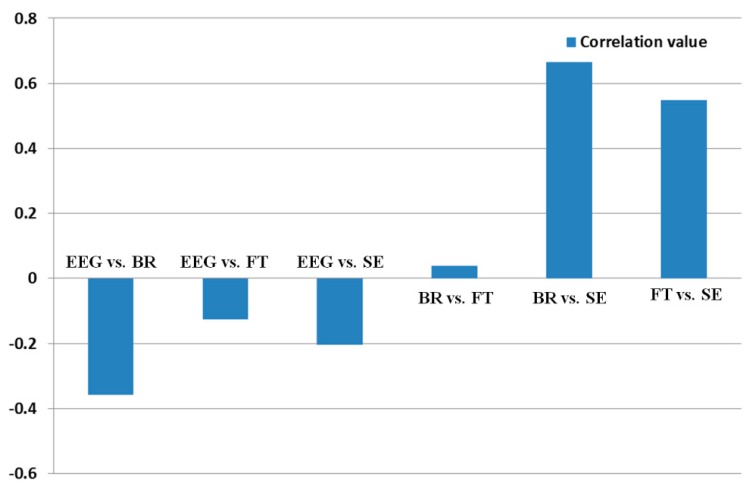
Calculated correlation value between each set of measurement.

The correlation value ranges from −1 to 1. Correlation values of −1 and 1 represent negative and positive relationships, respectively. If the correlation value is 0, it is almost uncorrelated. Because only the FT is reduced after watching the 3D display, as shown in Figure 12, whereas the SE, EEG, and BR increase as shown in Figures 9, 10 and 11, the FT data were multiplied by −1 in order to make them consistent. As shown in Table 8 and Figure 14, the absolute correlation value and R^2^ value between the BR and SE are greatest, and that between the BR and FT is lowest in the measurement of eye fatigue with a 3D display.

In order to simplify the comparison, we show the correlation matrix (based on the correlation value of Table 8) in Table 9. The correlation matrix of four sets of measured data (EEG, BR, FT, and SE) before and after (or in the last 1 min) watching the 3D display was calculated to enable the analysis of the statistical relationships [34].

As shown in Table 9, the relationships between the EEG and BR, EEG and FT, and EEG and SE were negatively related. Therefore, we find that the beta band of EEG data shows the inconsistent results with other data. This is because EEG signals contain noise that is caused by movements of the facial muscle. The relationships between the BR and FT, BR and SE, and FT and SE were positively related. As shown in Table 9, the absolute correlation value between BR and SE is highest, and that between BR and FT is lowest for the measurement of eye fatigue on the 3D display.

**Figure 15.**
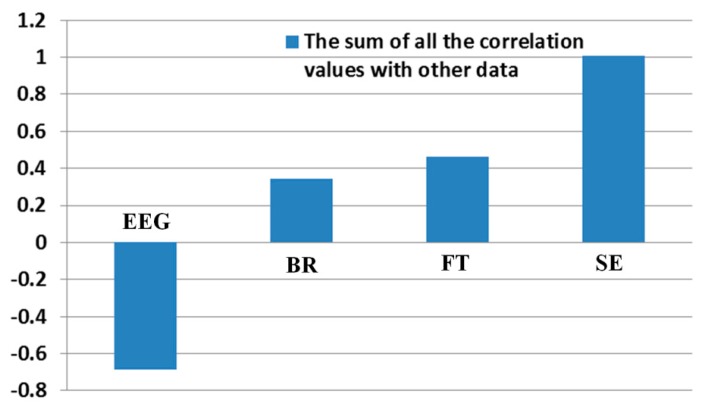
Calculated summed value of all the correlation values.

In order to quantitatively assess the individual correlations and the consistency of one data with others, we calculate the summed value of all the correlation values excluding the auto-correlation value of 1 (for example, correlation value between EEG and EEG). As shown in Table 9 and Figure 15, the correlation of SE with other data is highest based on the largest sum of 1.0092. In addition, those of the FT, BR, and EEG with other data are second, third, and fourth highest, respectively.

Previous researches showed that the level of eye fatigue increases after watching 3D displays [8–14], and there is no ground-truth value of the level of eye fatigue. Therefore, we measured the differences of four measurements of EEG, FT, BR, and SE score before and after (in the last one minute in case of BR) watching 3D display as the changed level of eye fatigue as shown in Tables 3, 4, 5 and 6 and Figures 9, 10, 11 and 12. However, because the units of each measurement value are not identical, we cannot regard the absolute difference value of each measurement as the difference of the level of eye fatigue. Therefore, we measured the difference between the amount of data before and after (or in the last one minute) watching the 3D display using the effect size in descriptive statistics as shown in Table 7. Based on Table 7 and Figure 13, we find that the difference between the SEs before and after watching the 3D display is largest. In addition, the differences between FT, EEG, and BR are second, third, and fourth largest, respectively.

However, if we measure the sum of correlation value of each measurement with others as shown in Table 9 and Figure 15, the correlation of SE with other measurement data is highest. In addition, those of the FT, BR, and EEG with other data are second, third, and fourth highest, respectively. From these, we find that the difference between the BRs before and in the last one minute for watching the 3D display is smaller than that between the EEGs as shown in Table 7, but the BRs are more credible than the EEGs considering the correlation with other data of Table 9. By conclusion, we find that the order of credibility of measurements for the eye fatigue on 3D display is SE, FT, BR, and EEG, respectively.

## 4. Conclusions

In this paper, we propose a novel assessment of eye fatigue caused by 3D displays based on a multimodal measurement method. In order to enhance the accuracy with which we assess the eye fatigue, we measured the changes in the EEG signal, eye BR, FT, and SE before and after (or in the last 1 min) of watching a 3D display. To accurately measure the eye BR in a manner that is convenient to the user, we implemented a remote gaze-tracking system using a high-speed mega-pixel camera that can measure the eye blinks of both eyes. The change in the FT is measured using a remote thermal camera that can enhance the measurement of eye fatigue. Experimental results showed that the correlation between BR and the SE score is highest, while that between BR and FT is lowest for the measurement of eye fatigue with a 3D display. In addition, the sum of all the correlation values of SE with other data (FT, BR, and EEG) is highest, and those of FT, BR, and EEG with other data are second, third, and fourth highest, respectively.

Because we aim at collecting accurate data of EEG, BR, and FT for the accurate measurement of the level of eye fatigue by non-intrusive way to user, our system is somewhat complicated as shown in Figure 2. In future work, we plan to simplify our capturing system. In addition, we would perform a more accurate evaluation of eye fatigue by combining the EEG signal, eye BR, FT, and SE based on approaches such as fuzzy rule or principal component analysis (PCA).

## Figures and Tables

**Table 1.** Summarized comparisons of previous and proposed methods used to measure eye fatigue.

**Category**		**Method**	**Advantages**	**Disadvantage**
Using single modality	Camera-based method [2–5]	Eye blink [2–5] and eye movement [3] were analyzed.	- Less discomfort to user than the bio-signal-based method because sensors not attached to body - Less affected by the movements of muscle, head, or body than the bio-signal-based method.	Lower acquisition speed of images than bio-signal-based method.
	Bio-signal-based method [6–12]	EOG [6], EEG [7–9,12], ECG [10,13], and blink signal from EEG [11] were analyzed.	Faster acquisition speed of data than camera-based method.	- More discomfort to user than the camera-based method because of attachment of sensors to body - More affected by the movements of muscle, head, or body than the camera-based method.
Using multiple modalities	Multiple bio-signal based method [14]	Multiple bio-signals such as ECG, GSR, and SKT were measured.	Higher accuracy of eye fatigue measurement than single modality-based method.	More discomfort to user because of attachment of multiple sensors, which can induce incorrect eye fatigue measurement.
	Hybrid method using both bio-signal and camera-based methods	EEG and BR were measured [15].	- Higher accuracy of eye fatigue measurement than single modality-based method. - Less discomfort to user than multiple bio-signal based method.	The accuracy enhancement of eye fatigue measurements using only two modalities (EEG and BR) is limited.
		EEG, BR, and FT were measured(**proposed method**).	- Higher accuracy of eye fatigue measurement than single modality-based method. - Less discomfort to user than multiple bio-signal-based methods. - Higher accuracy of eye fatigue measurement than [15] by using more modalities.	Additional thermal camera is required.

**Table 2.** Items in questionnaire form for SE.

**Six Questions for SE**
I have difficulties in seeing
I have a strange feeling around the eyes
My eyes feel tired
I feel numb
I feel dizzy looking at the screen
I have a headache

**Table 3.** The average and standard deviation of SE scores.

	**Before Watching 3D Display**	**After Watching 3D Display**
Average	1.623	3.478
Standard deviation	0.582	1.37

**Table 4.** The average and standard deviation of the EEG signal.

**Electrode**	**AF3**		**AF4**		**F3**		**F4**	
	Before	After	Before	After	Before	After	Before	After
Average	0.0921	0.1091	0.0916	0.1001	0.1059	0.1178	0.1033	0.1102
Standard deviation	0.0435	0.0353	0.0368	0.0362	0.0575	0.0526	0.046	0.0472
P-value	0.2491		0.5279		0.5575		0.6909	
Electrode	F7		F8		FC5		FC6	
	Before	After	Before	After	Before	After	Before	After
Average	0.0906	0.1165	0.0763	0.1118	0.1081	0.1086	0.0859	0.107
Standard deviation	0.0347	0.0431	0.0362	0.0823	0.0753	0.048	0.0403	0.0347
P-value	0.0814		0.1424		0.9812		0.1365	
Electrode	O1		O2		P7		P8	
	Before	After	Before	After	Before	After	Before	After
Average	0.1002	0.1159	0.1054	0.1218	0.0769	0.103	0.0984	0.1608
Standard deviation	0.0494	0.0438	0.064	0.0475	0.0369	0.0415	0.0466	0.1343
P-value	0.3662		0.4323		0.0795		0.1076	
Electrode	T7		T8					
	Before	After	Before	After				
Average	0.1037	0.1324	0.0954	0.0999				
Standard deviation	0.0668	0.0729	0.0568	0.0429				
P-value	0.2708		0.8071					

**Table 5.** The average and standard deviation of the eye BR.

	**Before Watching 3D Display**	**During the Last 1 Min of Watching 3D Display**
Average	18.667	22.6
Standard deviation	9.832	10.041

**Table 6.** The average and standard deviation of FT.

	**Before Watching 3D Display**	**After Watching 3D Display**
Average	15221.233	15099.446
Standard deviation	94.511	79.937

**Table 7.** The calculated Cohen's *d* values before and after watching the 3D display (In the case of the BR, the calculated Cohen's *d* before and in the last 1 min of watching the 3D display).

	**Cohen's *d***	**Effect Size**
EEG	0.6644	Large
BR	0.3958	Medium
FT	1.3914	Large
SE	1.763	Large

**Table 8.** The results of gradient, R^2^, and correlation between each set of acquired data.

	**Gradient**	**R^2^**	**Correlation**
EEG *vs.* BR	−0.33	0.1285	−0.3585
EEG *vs.* FT	−0.1154	0.0156	−0.125
EEG *vs.* SE	−0.1584	0.0421	−0.2052
BR *vs.* FT	0.0381	0.0014	0.038
BR *vs.* SE	0.5582	0.4427	0.6653
FT *vs.* SE	0.4593	0.3015	0.5491

**Table 9.** Correlation matrix of four measured data of before and after (or in the last one minute) watching 3D display.

	**EEG**	**BR**	**FT**	**SE**	**The Sum of All the Correlation Values with Other Data**
EEG	1	−0.3585	−0.125	−0.2052	−0.6887
BR	−0.3585	1	0.038	0.6653	0.3448
FT	−0.125	0.038	1	0.5491	0.4621
SE	−0.2052	0.6653	0.5491	1	1.0092
